# Optimization Strategies for Responsivity Control of Microgel Assisted Lab-On-Fiber Optrodes

**DOI:** 10.3390/s18041119

**Published:** 2018-04-06

**Authors:** Martino Giaquinto, Alberto Micco, Anna Aliberti, Eugenia Bobeico, Vera La Ferrara, Ruvo Menotti, Armando Ricciardi, Andrea Cusano

**Affiliations:** 1 Optoelectronics Group, Department of Engineering, University of Sannio, I-82100 Benevento, Italy; martino.giaquinto@unisannio.it (M.G.); alberto.micco@unisannio.it (A.M.); anna.aliberti@unisannio.it (A.A.); 2 ENEA, Portici Research Center, P. le E. Fermi 1, Portici, I-80055 Napoli, Italy; eugenia.bobeico@enea.it (E.B.); vera.laferrara@enea.it (V.L.F.); 3 Institute of Biostructure and Bioimaging, National Research Council, I-80143 Napoli, Italy; menotti.ruvo@unina.it

**Keywords:** lab-on-fiber, microgels, smart polymers, biochemical sensing

## Abstract

Integrating multi-responsive polymers such as microgels onto optical fiber tips, in a controlled fashion, enables unprecedented functionalities to Lab-on-fiber optrodes. The creation of a uniform microgel monolayer with a specific coverage factor is crucial for enhancing the probes responsivity to a pre-defined target parameter. Here we report a reliable fabrication strategy, based on the dip coating technique, for the controlled realization of microgel monolayer onto unconventional substrates, such as the optical fiber tip. The latter was previously covered by a plasmonic nanostructure to make it sensitive to superficial environment changes. Microgels have been prepared using specific Poly(*N*-isopropylacrylamide)-based monomers that enable bulky size changes in response to both temperature and pH variations. The formation of the microgel monolayer is efficiently controlled through the selection of suitable operating pH, temperature and concentration of particle dispersions used during the dipping procedure. The effect of each parameter has been evaluated, and the validity of our procedure is confirmed by means of both morphological and optical characterizations. We demonstrate that when the coverage factor exceeds 90%, the probe responsivity to microgels swelling/collapsing is significantly improved. Our study opens new paradigms for the development of engineered microgels assisted Lab-on-Fiber probes for biochemical applications.

## 1. Introduction

Lab-on-fiber (LOF) optrodes enable physical, chemical and biological measurements approaches where the interaction between the parameter to be measured and the light takes place either within the optical fiber itself (Lab-in-fiber) or within a structure which is totally integrated around (Lab-around-fiber) or onto the fiber tip (Lab-on-tip) [1,2]. Fiber tips, for example, provide a platform where it is possible to realize nanostructures, ranging from semiconductor photonic crystals to plasmonic devices compatible with the fiber dimensions, which precisely confine highly concentrated optical fields and facilitate the interaction of these fields with local physical, chemical and biological variations [3,4]. In this way, fiber-coupled spectroscopic measurements can be performed within the fiber optrode itself, providing information about superficial environment changes even at sub-wavelength scale [5,6]. Responsivity and sensitivity enhancement of LOF optrodes is achieved not only through the use of built-in sophisticated chemistry but also by integrating active compounds and advanced materials on the nanostructure [7]. In this respect, “smart” or stimuli-responsive polymers able to undergo large and reversible physical or chemical changes, may play a relevant role, especially when such changes occur in response to even small variations of external conditions, including temperature, pH, variations of the properties of surrounding light, variations of magnetic or electric field, variations of ionic factors, presence/absence of specific biomolecules, etc [8,9,10].

Recently, we have proposed a label-free biosensing platform based on the integration of microgels (MGs), i.e., micrometric hydrogel particles, with plasmonic LOF optrodes [5,11,12,13]. The MGs network, directly integrated onto the resonant nanostructure realized on the fiber tip, concentrates the target molecule and amplifies the optical response, leading to remarkable sensitivity enhancement for small molecule detection. We have also found that, by changing MGs concentration, it is possible to control the limit of detection, tune the working range as well as the response time of the probe, giving rise to advanced optrodes which can be easily reconfigured depending on the specific application [11]. With the aim of understanding the relationships between the MGs film properties and the device performances, we have also investigated and reported a numerical model able to describe the light-MGs interaction occurring on the resonant nanostructure [13]. In the wake of these results, it stands to reason that the combination of MGs with suitable chemical and physical features and specific LOF technologies could pave the way to a plethora of applications in the biomedical field, including drug delivery systems [14,15], cell culture supports [16], and sensors-actuators systems [17]. For this to become practical, the definition and acquisition of numerical and experimental tools for designing and realizing advanced MGs-assisted optrodes become a priority.

Techniques and protocols allowing the repeatable and controllable deposition of MGs on unconventional substrates such as fiber tips are however missing. Research groups have so far focused their efforts on understanding and manipulating the assembly of colloids only onto conventional planar surfaces. Glass, plastics and gold planar surface have been indeed coated with MGs film by employing several methods such as dip coating [18], spin coating [19,20], adsorption [21,22], drying from aqueous solutions [23,24], and centrifugation [25]. Depending on the method and process parameters, films with different particle density and homogeneity have been obtained. In this framework, Lyon and co-workers [26] reported a dip coating procedure followed by a covalent tethering step for the deposition and anchoring of MGs particles on poly(ethylene terephthalate) (PET) surfaces. The resulting MGs assembly was irregular in this pioneering work, nevertheless in other studies, the particles were densely packed to form a hexagonal lattice [18,24,27]. Highly ordered 2D Poly(*N*-isopropylacrylamide) pNIPAm-based films of colloidal crystals have been also prepared upon air-drying procedure by Kawaguchi and co-workers [23,28]. Authors claimed that a balance between capillary attraction and steric repulsion was the origin for the ordered regular distance between the particles in the film assembly. South et al. [25] reported a film fabrication approach that employed centrifugation (referred as “active” deposition) to assemble MGs films in an efficient, reproducible and fast manner. In this case, the high centrifugation speed segregated the hydrated particles onto a hard substrate, so that they were adsorbed onto small footprints and were more closely packed compared to particles passively deposited by simple adsorption. Recently, Serpe and coworkers [29,30,31] reported the so called “paint on” method that generated a dense, closely packed MGs film by continuously spreading and rotating the concentrated particles solutions on a gold planar surface. The quality of the MGs film was assessed by visual inspection, differential interference contrast (DIC) microscopy and by the high spectral purity observed upon optical analysis.

In the wake of these works, we have here investigated the feasibility of integrating MGs on patterned fiber tip with enhanced control of density distribution and coverage factor, through a dip coating procedure. We have evaluated the effects of MGs concentration, temperature and pH on the density of the particles on the fiber tip. The efficiency of deposition has been confirmed by means of both morphological analysis and optical characterizations. Finally, with a view towards sensing applications, coherently with our previous work [11], we demonstrate how different degrees of particle density and layer compactness give rise to different responsivities to MGs swelling/collapsing arising from both physical (temperature) and chemical (pH) parameters.

## 2. Materials and Methods

### 2.1. MGs Synthesis and Characterization

MGs used in our study were designed and prepared to make them simultaneously sensitive to temperature and pH. PNIPAm-based MGs, which are temperature responsive [17], were copolymerized with functional ionic monomers such as acrylic acid (AAc) to confer additional pH responsivity. The dual responsive PNIPAm-co-AAc MGs particles were synthesized following a standard precipitation polymerization method [32]. Specifically, polymerization was conducted in a 200 mL three-necked flask equipped with a condenser and a stirrer. A total of 0.900 g of *N*-isopropylacrylamide (NIPAM), 0.050 g of *N*,*N*′-methylene-bis-(acrylamide) (BIS), and sodium dodecyl sulfate (SDS solution 20% *w*/*v*, 25 µL) were dissolved in 98 mL of water and heated at the polymerization temperature of 70 °C under nitrogen purge for 1 h. An initiator solution made of 0.050 g potassium persulfate (KPS) in 1 mL of milliQ water was injected to initiate the polymerization. After 15 min, the AAc functional monomer (0.048 g in 1 mL of milliQ water) was added to the solution and the mixture left for 5 h under nitrogen to complete the co-polymerization reaction. After cooling, all MGs were cleaned by dialysis against deionized water for one week using tubes at 12–14 k nominal MWCO. The purified PNIPAm-co-AAc MGs were collected and lyophilized.

**Figure 1:**
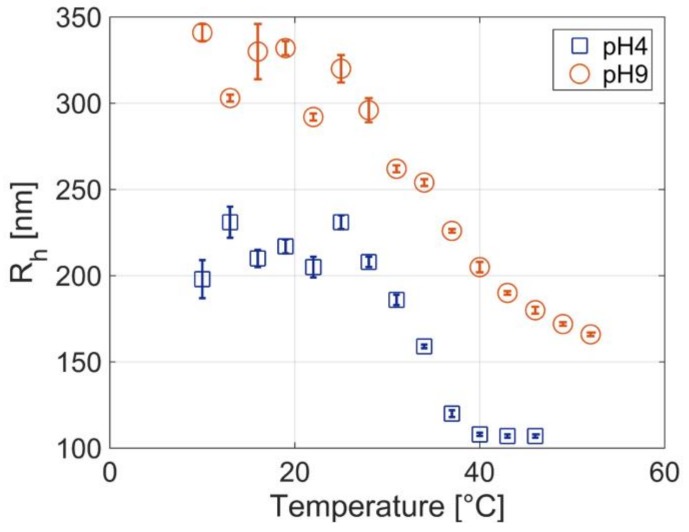
Dynamic Light Scattering (DLS) measurements of PNIPAm-co-AAc MGs at pH4 (blue squares) and pH9 (orange circles) as a function of temperature.

The PNIPAm-co-AAc MGs swelling behavior in response to temperature and pH was determined by means of Dynamic Light Scattering (DLS) measurements, evaluating the MGs hydrodynamic radii (R_h_). Measurements were carried out using a DLS system (Malvern Zetasizer Nano ZS instrument, 633 nm laser, 173° scattering angle, Malvern Instruments Ltd., Malvern, UK) supplied with a temperature controller. For thermo-responsivity measurements, an equilibration time of 1800 s was fixed for each temperature and a total of 5 runs were accumulated and averaged. The measurements were carried out in water at pH4, and in water at pH 9. pH was manually adjusted by using diluted HCl and NaOH solutions, respectively. As shown in Figure 1, PNIPAm-co-AAc MGs present a lower critical solution temperature (LCST) of ~34 °C in water at pH 4 (blue squares). At the LCST MGs have an entropically favored volume phase transition (VPT), since PNIPAm chains become hydrophobic and the interactions between them dominate expelling water and leading to a phase separation. Consistently, at lower temperatures, PNIPAm-co-AAc MGs have a R_h_ of ~210 nm that decreases to ~110 nm at higher temperatures.

Upon equilibration at pH 9 (orange circles), PNIPAm-co-AAc MGs exhibit an evident increase in curve breadth and size; in this case, the MGs R_h_ ranges from ~330 nm to ~170 nm by moving from low to high temperatures. This is due to the deprotonation of the AAc groups that by generating negative charges, increases the internal chain repulsion of MGs particles and the inter-particle repulsion forces. The resulting Coulombic repulsion and the increased osmotic pressure due to ion influx (Donnan potential) induce MGs swelling and size increase at alkaline pH.

### 2.2. LOF Probe Fabrication and Optical Characterization

The LOF probe is essentially formed by a metallic nanostructure integrated on the tip of a single mode optical fiber (9/125 SMF28, Corning Incorporated, New York, NY, USA). The probe was fabricated following an approach already described in our previous works [11,13]. A gold layer was deposited by e-beam evaporator system (Sistec KL400C, Kenosistec Srl, Binasco, Italy) on the fiber facet, preliminary cleaved with a precision cleaver (Fujikura CT-30, Fujikura Ltd., Tokyo, Japan). Successively, a square lattice of holes was drilled by a Focused Ion Beam (FIB) direct milling process (FEI Quanta 200 3D, Thermo Fisher Scientific, Waltham, MA, USA). The grating supports localized surface plasmon resonances which create a dip in the reflection spectra. The nanostructure geometrical parameters, namely the period, holes diameter, and gold thickness, were 850 nm, 306 nm, 50 nm, respectively.

The setup used for experimental measurements is essentially composed of a broadband optical source (NKT SuperK COMPACT, NKT Photonics, Birkerød, Denmarck), a 2 × 2 fiber optic coupler, and two Optical Spectrum Analyzer (OSA1: Ando AQ6315B, OSA2: Ando AQ6317C, Yokogawa Electric Corporation, Tokyo, Japan). Specifically, OSA1 measures the signal reflected by the sample in optrode configuration, while OSA2 measures the input signal. The reflected spectrum is given by the ratio between the data acquired from OSA1 and OSA2. Detailed description of the experimental setup can be found in Reference [11].

### 2.3. The Dip Coating Procedure

**Figure 2:**
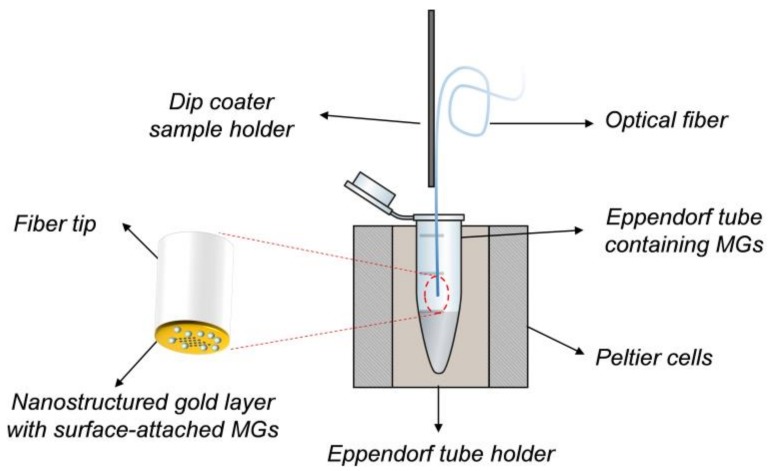
Schematic view of the setup used for microgels (MGs) deposition. In the zoomed image a detail on the gold nanostructure integrated on the fiber tip immersed in the MGs solution.

For integrating the MGs layer onto the fiber tip, we used the dip coating method (schematized in Figure 2). Specifically, by means of a dip coater (KSV NIMA KN4001, Biolin Scientific Oy, Espoo, Finland) the optical fiber probes were dipped into a 1.5 mL centrifuge tube containing a 500 µL aliquot of MGs solution for 1 h. Immersion and extraction speed was 5 mm/min.

During this phase, the temperature of MGs dispersion was kept constant by using a metallic centrifuge tube holder heated by Peltier cells-controlled system. The holder is basically composed of a brass die machined with a Computer Numerical Control (CNC) mill that matches perfectly the Eppendorf tube shape. The die is in contact with six Peltier cells (three for each side) that cool/heat the solution inside the tube. Two heat sinks are placed on the opposite face of the Peltier cells in order to dissipate the heat produced by the cells under the cooling phase, and thus keeping high the efficiency of the cells. A temperature range between 0 °C and 55 °C can be set inside the Eppendorf tube, when the surrounding environment is at room temperature.

Once the fiber is extracted from the MGs dispersion, the film was dried at 45 °C into an oven for 1 h. The optical fiber was then immersed in a deionized water bath for 12 h at room temperature under magnetic stirring in order to ensure breaking up of potential multilayers and their removal from the tip surface. Finally, the deposited MGs film was allowed to dry into an oven at 30 °C for 2 h.

### 2.4. Morphological Analysis

The MGs films created onto the fiber tips were morphologically characterized by means of an Atomic Force Microscopy (AFM) (Agilent Technologies 5420, Agilent Technologies, Santa Clara, CA, USA). AFM characterizations were performed directly onto the fiber tip in no-contact mode in order to prevent damage of the MGs film, by keeping fixed the fiber in a customized holder. The fiber holder basically consists of a steel block with a magnetic clamp that keeps the fiber vertically fixed in a groove. All the measurements were carried out when the MGs were in the *dry* state.

## 3. Results and Discussion

In the following, we report on the effect of all the degrees of freedom offered by MGs for achieving a uniform monolayer of closely packed particles onto the fiber tip. The scope is to develop a mix of these parameters leading to the creation of MGs film which guarantees the maximum degree of light matter interaction, and thus an optimization of the device performance. The strategy adopted in this work is based on our previous observations concerning the effect of MGs concentrations on the resulting film properties; larger concentrations give rise to films characterized by higher degrees of uniformity, density, and compactness. By taking this into account, here we first investigate the effect of temperature and pH (by keeping fixed the MGs concentration) for finding the optimum combination of parameters guaranteeing the maximum coverage factor. Successively, we exploit the third parameter, i.e., the MGs concentration for further improving the coverage factor.

Specifically, in this work, we tested two MGs solutions at fixed MGs concentration (0.5% *w*/*v*) prepared by using solution at pH 3 and pH 6 (pH adjusted by using diluted HCl and NaOH solutions), respectively. For each solution, two different deposition temperatures were investigated: 10 °C (below LCST) and 45 °C (above LCST). Successively two more MGs solutions at higher MGs concentrations (2% and 5%) were tested. By exploiting the above-mentioned deposition technique, different MGs-assisted LOF probes were fabricated starting from different MGs solutions, by modifying the properties of the deposition solution such as MGs concentration and pH. The results are presented and discussed in the following sections.

### 3.1. Effect of Temperature and pH

Temperature and pH are key parameters that can be varied to optimize the MGs deposition process. At low temperatures (below LCST), MGs are highly hydrophilic and swell in water by virtue of hydrogen bonds between MGs and water molecules. On the other hand, at high temperatures (>LCST), MGs are collapsed and a partial aggregation is favored due to MG-MG hydrophobic interactions. Therefore, by tuning the deposition temperature, it could be possible to control the MG-MG interaction and thus the density of the film layer. Moreover, the effect of the pH on MGs assembly behavior is a result of a complex balance between MG-MG and MG−surface interactions. At low pH (i.e., pH 3), almost all AAc groups present in the MGs network, are protonated, the particles are more or less uncharged, and the formation of a closed packed film is favored. At higher pH (pH 6) instead, a partial dissociation of carboxylic acid groups is achieved, MGs are negatively charged, and the electrostatic MG-MG repulsion leads to a consequent package density decrease.

**Figure 3:**
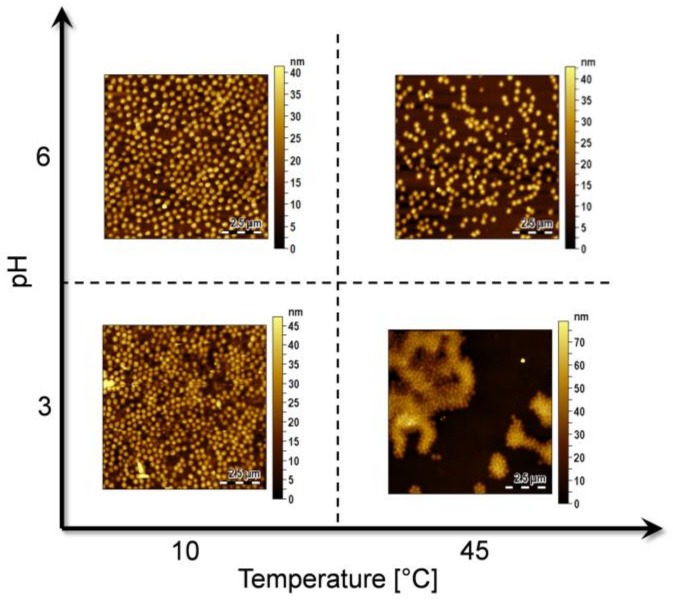
MGs films realized on the optical fibers tip by exploiting the effect of temperature and pH, keeping fixed the concentration at 0.5%. For each couple of temperature and pH an area of 10 × 10 µm above the fiber core region is shown.

As a consequence, both the temperature and pH of the MGs solution dramatically affect the final MGs film properties, in terms of uniformity of distribution and compactness. Figure 3 shows the AFM images measurements carried out on an unpatterned area of 10 × 10 μm onto the fiber tip, pertaining to probes exposed to MGs solutions at pH 6, at 10 °C (top-left corner), pH 6 at 45 °C (top-right corner), pH 3 at 10 °C (down-left corner) and pH 3 at 45 °C (down-right corner). In this first analysis, the MGs concentration was 0.5%, in line with our previous works [11,13].

Looking at Figure 3 we observe that the film prepared at pH 3 at T = 10 °C shows a higher packing density than that prepared at the same temperature but different pH (pH 6). This is due to a pH-induced perturbation of the forces regulating the MGs film formation. In fact, as already shown in a previous work [29], two opposing forces control the distance between the particles, and consequently, the morphology of MGs film: first, MG-MG interactions and second, coordination of the surface and particles. MG−MG interactions comprise soft repulsive interactions and attractive interactions (van der Waals interactions, hydrophobic interactions, inter/intramolecular hydrogen bonding) [27]. The interactions between MGs and the surface are instead the result of the strong coordination between nitrogen and oxygen atoms, present in the MGs polymeric network, and gold [33,34]. The Au-MGs bond is also promoted by weak van der Waals interactions and by lone pair present on the amide nitrogen occurring in NIPAM [29].

These considerations well explain the highest packing density of the film prepared at pH 3 and at low temperature (10 °C) in Figure 3. In this case, almost all AAc groups are protonated and the strong MG-surface interaction prevails on the low MG-MG electrostatic repulsion. Close-packing of the particles at pH 3 is also promoted by inter-particle attractive hydrogen bonding between the protonated AAc side chain groups and the amide group of NIPAM; these interactions can occur only with the neutral form of the carboxylic moiety [19]. During deposition at pH 6 and at 10 °C, the MGs are negatively charged and the assembly of MGs on the Au surface is frustrated by the strong Coulombic repulsion resulting in fewer MGs attached to the Au surface with a higher particle-particle distance (see Figure 3). In this case, the contribution of MGs and surface interactions has a weak effect in comparison to MG-MG interactions.

The films deposited at pH 3 at 45 °C is less homogeneous than the corresponding films deposited at 10 °C, and a strong MGs aggregation is observed. In general, MGs films deposited at temperatures above the LCST present a high degree of heterogeneity, due to hydrophobic aggregation of MGs during deposition. In fact when MGs are shrunk, they tend to aggregate due to hydrophobic interactions [19] and, consequently, the resulted films are less homogeneous.

Similar considerations also hold for the film obtained at pH 6 and at higher temperature, which is characterized by a particle density smaller than that obtained in the film prepared at 10 °C. In this case, the low number of particles is affected by Coulombic repulsions existing at pH 6 and due to the partial deprotonation of the acrylic groups. We also hypothesize that Brownian motions, that are strongly dependent on temperature, could affect the density of the MGs layer, causing a partial rarefaction of adsorbed MGs.

Overall, from Figure 3 it results that the highest MGs density on the fiber tip is obtained with low temperatures and low pHs. Following this trend, a further decrease of temperature or pH would provide higher MGs densities. However, as shown by DLS measurements (see Figure 1), MGs are not sensitive to temperature changes below about 30 °C, thus a decrease below 10 °C is not expected to cause any further change in the deposition. Similarly, since at pH 3 we are below the pKa of AAc (pKa ≈ 4.8), all carboxylic groups are protonated, a further reduction of the pH value will not affect that much particle density and film deposition.

### 3.2. Effect of MGs Concentration

**Figure 4:**
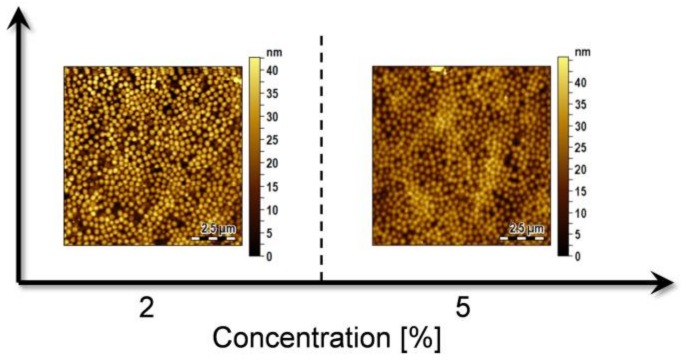
MGs films realized on the optical fibers tip at two different concentration, 2% and 5%, at pH 3 and at 10 °C. For each concentration an area of 10 × 10 µm above the fiber core region is shown.

By keeping constant the optimal pH and temperature conditions previously identified (pH 3, T = 10 °C), we next investigated the effect of particle concentration on the compactness of the film. We have already shown in a previous work that MGs concentration strongly affects the film morphology and packing density of the deposited layer [13]. Therefore, the concentration of the MGs dispersion was increased from 0.5% to 2% and 5%. As expected, the density of deposited MGs particles increases with increasing particle concentration (Figure 4) and at the concentration of 5%, the MGs film was mostly homogeneous.

### 3.3. Evaluation of the Coverage Factor

**Figure 5:**
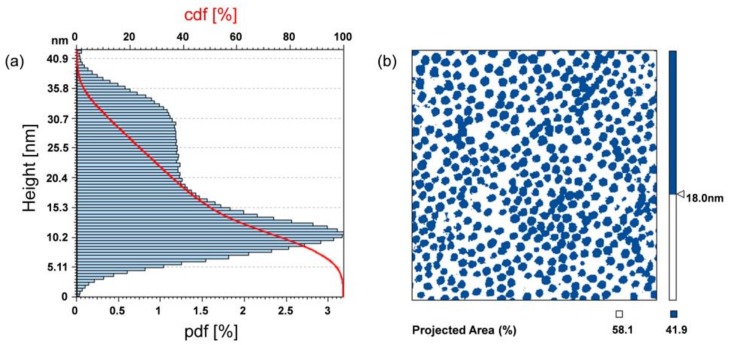
(**a**) Atomic Force Microscopy (AFM) measured points pdf (blue-bars) and cdf (red curve) of the profile height for the MGs film obtained with T = 10 °C and pH 6. (**b**) Substrate region covered by MGs, defined with a slice process in correspondence of 18 nm.

With the aim of quantitatively evaluating the differences between the MGs films realized under different conditions, more detailed analyses of the AFM data were carried out. Firstly, we estimated the coverage factor, i.e., the rate of Au substrate covered by MGs, by processing the measured AFM profiles. The analysis consisted in identifying the area of MGs projected on a plane parallel to the Au substrate. Since the Au substrate is characterized by a certain roughness, and the surface attached MGs cross-section area is a decreasing function of their height, the threshold for the estimation of the coverage factor needs to be accurately defined. In Figure 5, for the specific case of T = 10 °C and pH 6, the measured point probability density function (pdf) of the profile height (i.e., the profile trace) is shown. In the same figure, the Abbot-Firestone curve is also shown in red, which represents the cumulative probability density function (cdf) of the profile height and can be calculated by integrating the profile trace.

From the profile trace, we observe the presence of two peaks, describing the point distribution associated to the Au substrate roughness (with the maximum value assumed at around 10 nm), and the point distribution associated to the MGs profile (with the maximum value assumed at around 30 nm). The height corresponding to the intersection between the two distributions (~18 nm) was chosen as threshold for the coverage factor definition. In fact, the points above a plane parallel to the gold substrate passing for the intersection height defines the regions covered by MGs, while the points below this plane define the uncovered regions (i.e., the bare Au substrate). This concept is schematically shown in Figure 5b, where the covered regions are represented in blue, while the uncovered regions in white. The coverage factor essentially corresponds to the Abbot-Firestone curve value assumed at 18 nm. The illustrated procedure was applied also to the other cases studied, obtaining the results reported in Table 1.

To assess the reliability of the deposition procedure, the coverage factors were evaluated as the mean value on 5 depositions realized for each couple of temperature and pH values. The absolute uncertainty (corresponding to one standard deviation), which is less than 5% in most of the cases reported in Table 1, essentially demonstrates that the deposition procedure is reliable and repeatable.

### 3.4. Optical Characterization and Responsivity Analysis

As previously mentioned, controlling the coverage factor of the MGs monolayer, and in particular achieving a well-packed and dense particles film onto the resonant fiber tip, enables the maximum degree of light-MGs interactions. Consequently, the MGs-assisted LOF optrode responsivity to MGs swelling/collapsing induced by external physical and chemical stimuli is strongly enhanced. As a further confirmation of this concept and to highlight the significance of the coverage factor control in view of potential sensing applications, we evaluated the responsivity to such changes. As a case study, we analyzed two probes characterized by the minimum (21%) and maximum (92%) MGs coverage factors.

**Figure 6:**
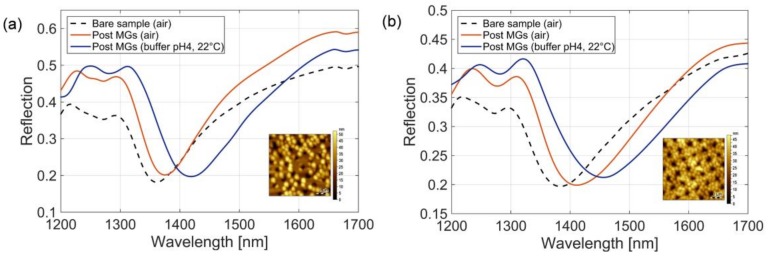
Experimental spectra evaluated before MGs deposition in air, and post MGs deposition, both in air, and buffer solution (pH 4, 22 °C), of the sample 1, with coverage factor of 21% (**a**) and sample 2, with coverage factor of 92% (**b**). Insets in (**a**,**b**) show the MGs attached on the patterned optical fiber tip obtained by means of AFM measurements.

Measured reflection spectra of probes pertaining to the MGs depositions made at (i) T = 45 °C, pH 6, 0.5% and (ii) T = 10 °C, pH 3, 5% are shown in Figure 6a,b, respectively. Reflection spectra in air before and after the MGs deposition are shown as black dotted and red solid curves, respectively, while the blue curves correspond to spectra of probes with MGs in buffer solution at pH 4 and at 22 °C.

After MGs deposition, the reflection dip of the probe with the coverage factor of 21% underwent a red-shifts of 14 nm and 61 nm when the probe was in air and in solution at pH 4 and at 22 °C, respectively. On the other hand, the reflection dip of the probe with larger coverage factor shifted by 35 and 73 nm in the same conditions. The wavelength shift between dashed black curve (initial spectrum) and the red curve spectra is due to the MGs deposition, i.e., to a *local* refractive index change. Moreover, the resonant wavelength shift between blue and red curve spectra are due to both *local* refractive index changes (induced by the MGs swelling), and the *bulk* refractive index change (induced by the transition from air to the buffer solution). Consistently with previous observations [11], the larger was the MGs coverage factor, the higher was the resonance shifts measured in the dry conditions. The equivalent refractive index due to a denser particles distribution clearly resulted in a larger shift. Specifically, the larger MGs coverage factor (92%) leaded to a wavelength shift enhancement of a factor ~2.5 with respect to the low coverage factor counterpart. In wet conditions, and in the totally swollen regime at 22 °C, the density of the MGs layer did not show a strong effect on the bulk sensitivity. In fact, wavelength shifts of 61 and 73 nm were measured for sample 1 and sample 2 respectively, meaning that MG coverage factor of 92% leaded to a wavelength shift enhancement of about the 20% (in dry conditions it was 150%). This is likely due to the fact that when MGs are completely swollen, the equivalent refractive index of the resulting layer is very close to that of the buffer solutions in both the cases. It is interesting to note that when the optical probes integrated with MGs are dipped in the buffer solution, the measured wavelength shifts were 47 nm (14 nm to 61 nm) and 38 nm (35 nm to 73 nm), for the sample 1 and sample 2, respectively. This is essentially due to the fact the high MGs particle coverage (i.e., more polymer component on surface) makes the liquid effect less prominent.

**Figure 7:**
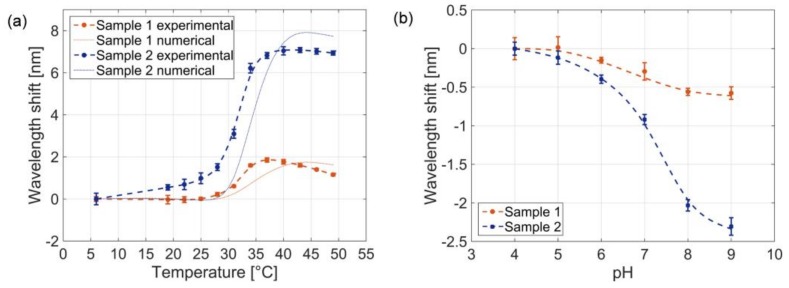
Resonant wavelength shift as function of solution temperature at pH 4 (**a**) and as function of the solution pH at 32.5 °C, of the sample 1, with coverage factor of 21% (**a**) and sample 2, with coverage factor of 92% (**b**). The error bars represent one standard deviation evaluated on 5 acquisitions. The dashed lines were obtained with a smoothing spline fitting.

**Figure A1:**
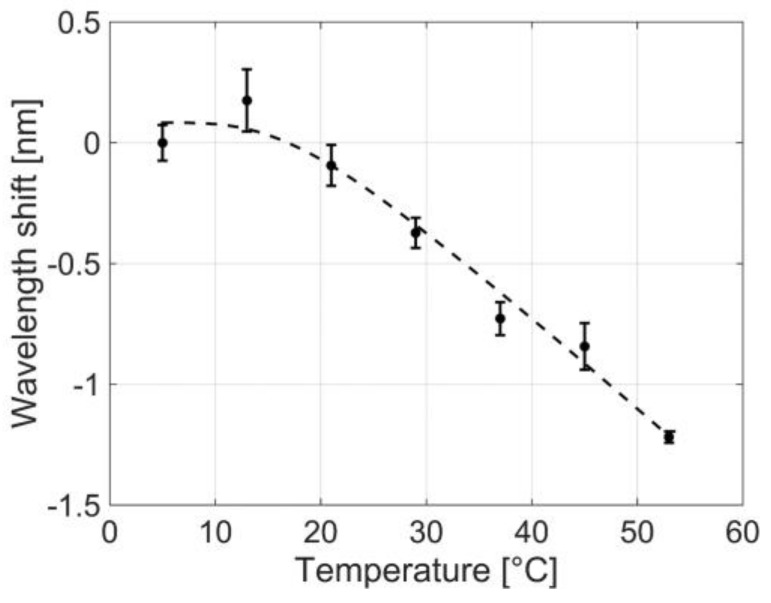
Resonant wavelength shift of the bare sample (without MGs) as function of water temperature. The dashed line was obtained with a smoothing spline fitting.

Successively, we study the temperature responsivity of the two probes. Figure 7a shows the evolution of the resonant wavelength (corresponding to the reflectance dip) as a function of temperature at pH 4. Resonant wavelengths were obtained as ‘central wavelength’, i.e., by evaluating the power weighted mean wavelength of the reflection spectrum. Resonant wavelengths reported in Figure 7 are the average value over 5 measurements taken at each temperature/pH value. As expected, we notice that the larger resonant wavelength shift excursion is achieved with probes pertaining to the larger coverage factors. Specifically, the maximum wavelength shifts induced by temperature variations were 2 nm and 7 nm for sample 1 and sample 2 respectively, so that a temperature sensitivity enhancement of a factor 3.5 is achieved with high MGs coverage factor. The measured shifts are directly related to the MGs swelling/collapsing dynamics. Furthermore, the decreasing trend of the resonant wavelength in the high temperature range (particularly evident for sample 1), where MGs do not change their size, is due to the negative thermo-optic effect induced by the buffer solution in which the probes are immersed. In fact, temperature sensitivity dλ/dT of bare LOF probe, i.e., without MGs layer, was measured to be −35 pm/°C in the range 10–50 °C (see Figure A1). Moreover, it is interesting to observe that the total shift of 2 nm, achieved with MGs coverage factor of 21%, is comparable to that achieved by using the LOF probe without MGs integration (see Figure A1).

Results clearly demonstrate that the entity of the wavelength shift induced by temperature variations is clearly related to the MGs density on the optical fiber tip surface. However, a rigorous correlation and the definition of a correlation factor between the overall wavelength shift and the MGs coverage factors is not trivial as it could not be linear; in fact, the wavelength shift is the result of different physical effects including thermo-optical, MGs particle properties, and the electromagnetic field distribution of the plasmonic resonance.

**Figure A2:**
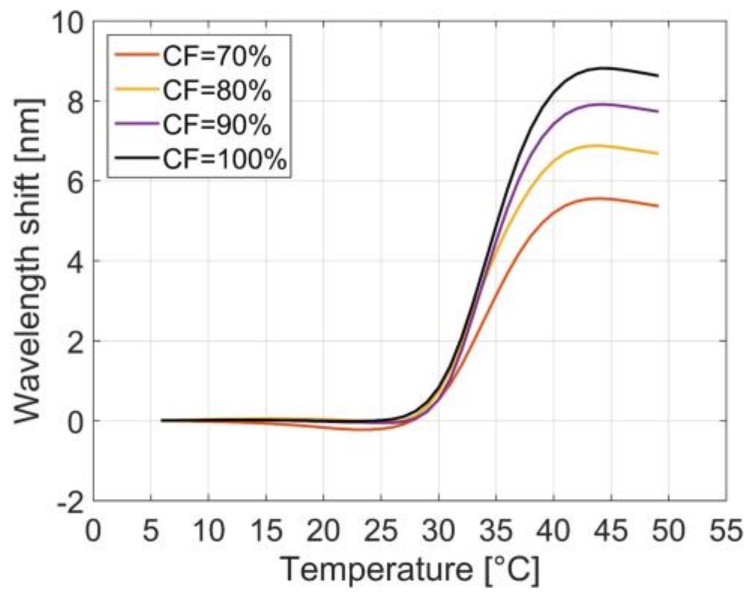
Numerical resonant wavelength shift as a function of the MGs slab thickness at different coverage factors.

The experimental wavelength shift as function of temperature is in good agreement with the trend evaluated through the model described in our previous work [13]. In fact, the wavelength shift induced by the MGs swelling/collapsing is mainly determined by the resulting layer refractive index, whose value variations become negligible when the MGs coverage factor is low. In our model we have taken into account the thermo-optical effect derived from results reported in Figure A1. A more exhaustive discussion on the relationship between microgel size (DLS) and wavelength shifts (optical characterizations) has been provided in Reference [11]. Coverage factors larger than 90% (difficult to achieve with a single dipping procedure), would have given rise to slightly larger total wavelength shifts, since the equivalent refractive index of the slab formed by the MGs film formed on the gold layer tends to saturate for these coverage factor values (see Figure A2).

Finally, we also carried out experimental measurements for evaluating the pH responsivity. To this aim, the probes were dipped in a pH 4 buffer solution at 32.5 °C, at the middle of the transition curve. Successively, the pH of the buffer solution was increased up to pH 9 by adding diluted NaOH. Reflection spectra were measured at each pH value and the corresponding resonant wavelengths (reflection spectra dips) are shown in Figure 7b. In agreement with the previously discussed observations, the probe with a denser MGs film responded with a larger wavelength shift (2.3 nm). Figure 7 essentially demonstrates that MGs-assisted probes have a ‘qualitatively similar’ response to both temperature and pH changes. For temperatures smaller than 25 °C or higher than 35 °C the MGs VPT vanishes and tend to saturate to a specific value. Similar considerations hold for MGs response induced by pH variations, where the transition occurs in the range pH 4–9. The optical sensitivity of the probes is not linear within the investigated pH range. As anticipated in Section 2.1, this is most likely due to the tendency to saturate of MGs response in response to pH as it reaches the most extremes values. However, increase and tuning of the linearity range of the pH responsivity can be achieved by acting on the functional acrylic monomer chemical structure, i.e., by using AAC monomer with alkyl chain of different length attached to the second carbon [35] or by manipulating different cationic monomer as amino-acrylate [36].

The MGs, being intrinsically multi-responsive polymers, may respond to both temperature and pH changes at the same time. In this paper, for validating the proposed deposition technique, we have evaluated the temperature response by keeping fixed the pH, and vice versa. In any case, the cross sensitivity can be overcome by making the pH characterization at specific temperature ranges where MGs are temperature insensitive. Moreover, MGs can be synthesized by using appropriate sets of monomers, which make them completely insensitive to temperature, but only responsive to pH. Similarly, by preparing MGs with a cross-linker different from AAc so, they do not respond to pH changes.

Overall, the optical characterizations reported in this section confirm that the previous discussed deposition procedures enable an effective control of the MGs particles coverage, and consequently the capability to fully control the probe responsivity to a particular parameter of interest. A discussion on sensitivity enhancement approaches by acting at both nanostructure and MGs typology level goes far beyond the scope of this work.

## 4. Conclusions

In conclusion, we have proposed a fabrication procedure for integrating in a controlled fashion multiresponsive MGs onto optical fiber tips. The method allows the deposition of an active monolayer with a specific coverage factor, preventing particles aggregation, which mostly lead to weak process repeatability and random response. Through both morphological and optical analyses, we found that, by acting on the combination of temperature and pH it is possible to achieve coverage factors in a range varying between 20% and 75%. Next, we found that optimal coverage factors (>90%) can be achieved by further increasing the MGs concentration in the deposition solution used during the dip coating procedure. In fact, achieving coverage factors larger than 90% warrants the maximum degree of light-MGs interactions onto the fiber tip, and thus the maximum responsivity to MGs swelling/collapsing induced by the specific external stimulus of interest.

Without loss of generality, the validity of our process was applied to optical fiber integrated with a metallic nanostructure supporting localized surface plasmon resonances. Although this is here demonstrated with a specific set of MGs and one single substrate material (gold), the proposed protocol could be likely also extended to different multiresponsive MGs possibly integrated on different typologies of lab on tip platforms (dielectric nanostructures). The influence of other process parameters such as immersion and drying time, surfactant concentration, etc. have not been investigated and will be the object of future works. Once assessed the optimization strategies enabling the responsivity control of MGs-assisted optrodes, our results will pave the way to the development of advanced platforms, which, thanks to the multiresponsive MGs integrations, become very interesting systems, especially from a biomedical point of view. Indeed, highly responsive MGs-assisted LOF optrodes, possibly integrated inside medical needles and catheters, will be consolidated in the coming years, eventually becoming a unique platform for performing multifunctional operations such as operations such as detection and delivery [9,10,14,15,17] of molecules, such as drugs, directly inside the human body. It is worth noticing in this context that thermosensitive MGs exhibit transition temperatures close to physiological values. Moreover, pH responsivity has the potential application to deliver environment-guided responses into specific areas of the human body characterized by significant pH variations such as the gastrointestinal tract or tissue compartments affected by specific tumoral features [37].

## Figures and Tables

**Table 1:** Coverage factors obtained at different temperatures, pHs and MGs concentrations. The absolute uncertainty corresponds to the standard deviation evaluated over 5 samples.

MGs Concentration	Solution Temperature (°C)	Solution pH	Coverage Factor (%)
5.0%	10	3	91.8 ± 1.6
2.0%	10	3	81.7 ± 2.3
0.5%	10	3	74.5 ± 4.6
0.5%	10	6	40.0 ± 4.2
0.5%	45	3	33.7 ± 5.7
0.5%	45	6	21.3 ± 4.8
